# Ancient DNA Analysis Reveals High Frequency of European Lactase Persistence Allele (T-13910) in Medieval Central Europe

**DOI:** 10.1371/journal.pone.0086251

**Published:** 2014-01-23

**Authors:** Annina Krüttli, Abigail Bouwman, Gülfirde Akgül, Philippe Della Casa, Frank Rühli, Christina Warinner

**Affiliations:** 1 Centre for Evolutionary Medicine, Institute of Anatomy, University of Zurich, Zurich, Switzerland; 2 Department of Pre- and Protohistory, Institute of History, University of Zurich, Zurich, Switzerland; 3 Department of Anthropology, University of Oklahoma, Norman, Oklahoma, United States of America; Natural History Museum of Denmark, Denmark

## Abstract

Ruminant milk and dairy products are important food resources in many European, African, and Middle Eastern societies. These regions are also associated with derived genetic variants for lactase persistence. In mammals, lactase, the enzyme that hydrolyzes the milk sugar lactose, is normally down-regulated after weaning, but at least five human populations around the world have independently evolved mutations regulating the expression of the lactase-phlorizin-hydrolase gene. These mutations result in a dominant lactase persistence phenotype and continued lactase tolerance in adulthood. A single nucleotide polymorphism (SNP) at C/T-13910 is responsible for most lactase persistence in European populations, but when and where the T-13910 polymorphism originated and the evolutionary processes by which it rose to high frequency in Europe have been the subject of strong debate. A history of dairying is presumed to be a prerequisite, but archaeological evidence is lacking. In this study, DNA was extracted from the dentine of 36 individuals excavated at a medieval cemetery in Dalheim, Germany. Eighteen individuals were successfully genotyped for the C/T-13910 SNP by molecular cloning and sequencing, of which 13 (72%) exhibited a European lactase persistence genotype: 44% CT, 28% TT. Previous ancient DNA-based studies found that lactase persistence genotypes fall below detection levels in most regions of Neolithic Europe. Our research shows that by AD 1200, lactase persistence frequency had risen to over 70% in this community in western Central Europe. Given that lactase persistence genotype frequency in present-day Germany and Austria is estimated at 71–80%, our results suggest that genetic lactase persistence likely reached modern levels before the historic population declines associated with the Black Death, thus excluding plague-associated evolutionary forces in the rise of lactase persistence in this region. This new evidence sheds light on the dynamic evolutionary history of the European lactase persistence trait and its global cultural implications.

## Introduction

Lactase persistence (LP) is a common genetic trait in many European, African and Middle Eastern populations. In Europe, LP primarily results from a C→T transition located approximately 14,000 bp upstream of the lactase-phlorizin-hydrolase (*LCT*) gene in intron 9 of the minichromosome maintenance 6 (*MCM6*) gene on chromosome 2 [1], [2]. A thymine at this locus (T-13910) prevents down-regulation of lactase activity after weaning. In addition to the European C/T-13910 LP SNP, this region also includes four other SNPs associated with LP in Arab (T/G-13915) and African (C/G-13907, T/C-13913, G/C-14010) populations [2]–[4].

LP is an autosomal dominant trait, such that only homozygous wildtype individuals cease lactase production after childhood [1]. The enzyme lactase hydrolyses the milk disaccharide lactose into its component monosaccharides, galactose and glucose for absorption in the small intestine. If it is absent, the lactose cannot be absorbed by the intestinal mucosa and therefore reaches the colon undigested where it is fermented by colonic bacteria, often causing symptoms such as abdominal pain, bloating and diarrhea [5]. Modern frequencies of lactase persistence vary throughout Europe according to a geographic cline. They are highest in northwestern Europe, gradually declining towards the southeast [6]. In western Central Europe (Germany and Austria), reported LP genotype (CT-13910 and TT-13910) frequencies range from 71–79.8% [7]–[11]. When and where the LP T-13910 polymorphism originated and the evolutionary processes by which it became the majority allele in Europe have been the subject of strong debate [12]–[24]. Because milk is the only natural source of lactose, it is thought that a culture of dairying must correlate with lactase persistence to some extent.

Two hypotheses have been proposed to explain the origin of LP and its association with dairying: the culture-historical hypothesis [25] and the reverse cause hypothesis [26]. The culture-historical hypothesis posits that the LP allele arose from low frequency through selection in cultures with a long history of dairying. By contrast, the reverse-cause hypothesis holds that the LP allele may have already been common in certain populations due to genetic drift and only these populations would have adopted the cultural practice of dairying. Additional genetic pressures may have existed in arid climates where milk is one of the only clean sources of water [27] or in northern latitudes where, in the absence of vitamin D, the presence of lactose facilitates the absorption of calcium by the intestinal mucosa and thus reduces the risk of rickets and osteomalacia [28]. Rickets and osteomalacia can cause deformation of the pelvis and are leading causes of obstructed labor and consequent maternal mortality and perinatal morbidity in traditional societies without access to modern medical care [29].

Keeping cattle, sheep and goats not only for meat but also for milk provided important advantages for ancient societies. Milk contains high quality fat, protein and sugar and high amounts of calcium [30]. It is a clean liquid that can be converted into a variety of storable products that may have helped prehistoric people survive periods of scarcity [17]. It has been estimated that a prehistoric cow was able to produce 400–600 kg of milk in a lactation period of three to four months. Even after subtracting the 250–350 kg needed for the raising of a calf, 150–250 kg of milk would remain available for human consumption. The caloric content of this milk is almost equivalent to the meat of an entire cow, and it can be exploited multiple times throughout the animal's lifetime [31].

Although archaeological evidence for dairying in prehistoric Europe is scarce, recent zooarchaeological and biomolecular data suggest that dairying was practiced from the very beginning of the Neolithic (ca. 5000 BC) and became more and more important over time [16], [23], [32]. Another line of evidence comes from SNP [13] and microsatellite variation [33] studies that report strong signals of recent positive selection at the C/T-13910 locus. These studies estimate that selective pressure on the allele began 2188–20650 years BP and 7450–12300 years BP, respectively, and computer simulations place the origin of the allele at 6256–8683 years BP in a region between the central Balkans and Central Europe [18].

**Figure 1:**
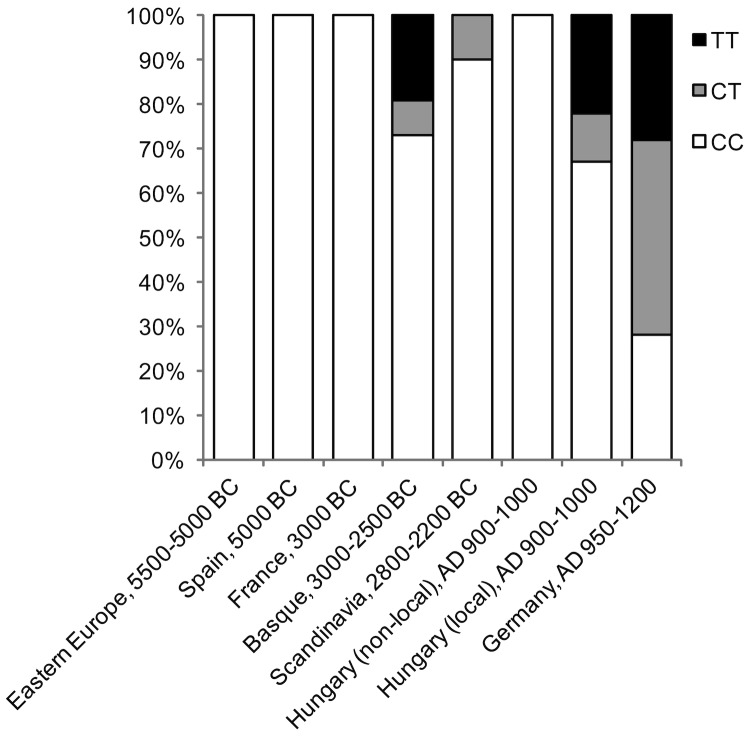
Relative frequency of lactase persistence and non-persistence genotypes reported in past European populations. CC: lactase non-persistent (wild type); CT and TT: lactase persistent. Study sample sizes and references are provided in Table 3. Data for Germany (AD 950–1200) are from this study.

Previous ancient DNA studies of LP have reported low, but highly variable frequencies for the lactase persistence allele in Neolithic Europe (Figure 1). In a Neolithic Basque population the lactase persistence allele was found in seven out of 26 individuals, of which two were heterozygous and five were homozygous for the LP allele [34]. In a separate study, one Neolithic individual from Sweden was also found to be heterozygous [35]. All other studies reported only homozygous lactase non-persistent genotypes [14], [36], [37]. To date, only one published ancient DNA study has investigated LP prevalence in a post-Neolithic population; in that study on medieval Hungary (ca. AD 900–1100), local commoners and foreign conquerors were found to have different LP genotype frequencies [38]. The foreign conquerors (originating from the east) were found to have exclusively lactase non-persistent genotypes, whereas the local commoners exhibited 33% LP genotypes (CT and TT). As modern LP genotype frequencies in the region are >60% today [38], these results imply rapid allele frequency change during the past millennium.

Many questions remain regarding the evolution of LP in prehistoric and historic Europe. In order to understand this process in more detail, we investigated a medieval (ca. AD 950–1200) cemetery located in the village of Dalheim in Nordrhein-Westfalen, Germany [39] and successfully genotyped 18/36 individuals for LP alleles. We demonstrate that the frequency of the LP in this western Central European community, had already reached modern levels by AD 1200, and we report the highest LP T-13910 allele frequency determined to date for any ancient population.

## Results

MtDNA was successfully amplified for 31/36 individuals (86%). The five individuals yielding poor or inconsistent sequences and were excluded from further analysis. Genetic sex typing was successful for 27 of the remaining individuals, and of these, 18 were also successfully genotyped for LP SNPs.

**Figure 2:**
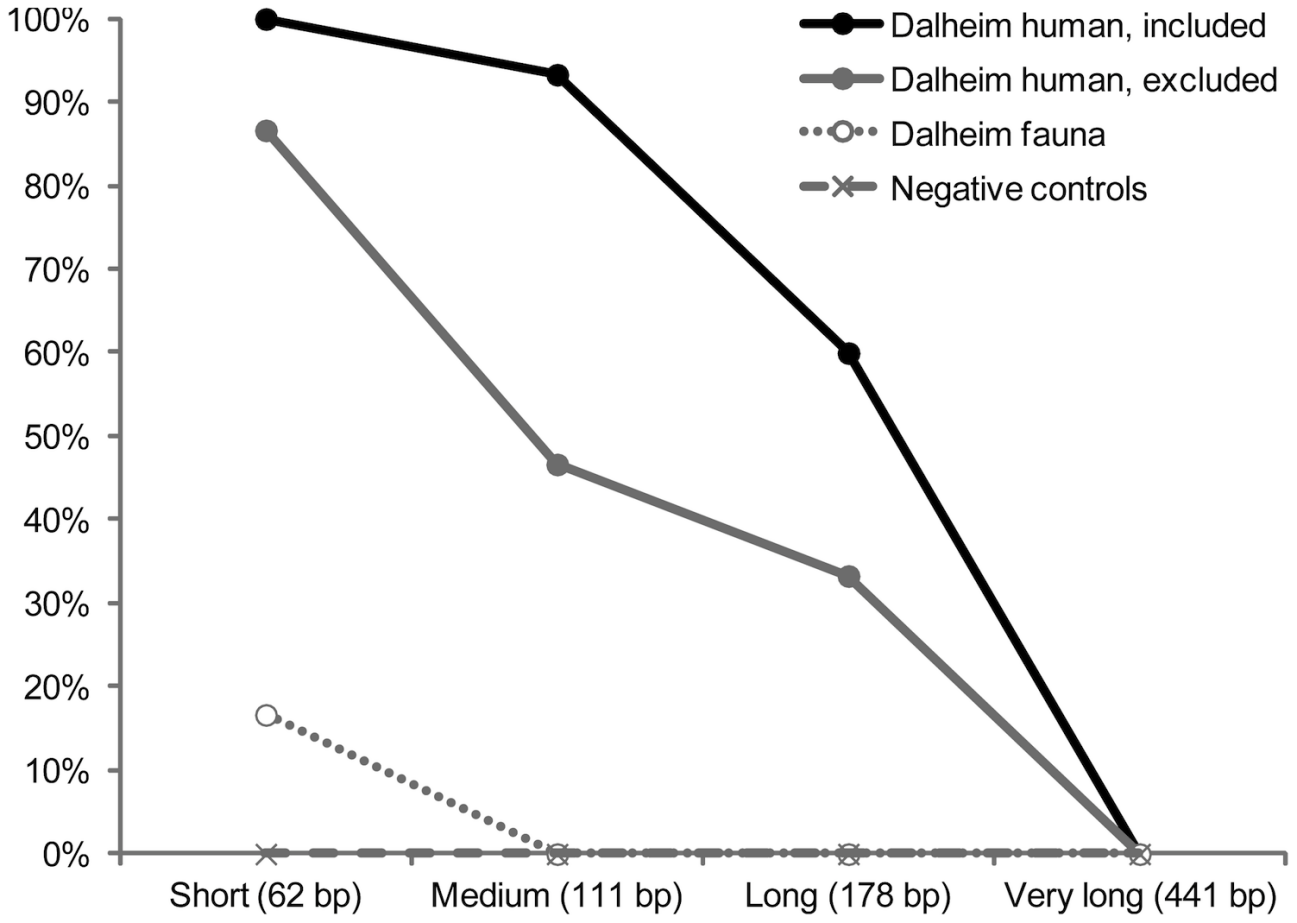
Results of ancient DNA authentication test of Dalheim human and control samples as determined by real-time PCR. Well preserved Dalheim human samples included in this study show strong evidence of asymmetrical molecular behavior consistent with ancient DNA. Poorly preserved Dalheim human samples also show evidence of asymmetrical molecular behavior, but they were excluded from further analysis because their poor amplification success rate (<50% a the 111 bp length used for genotyping) makes them vulnerable to amplification bias and allelic dropout. Control samples show no or spurious amplification. Sample sizes are as follows: Dalheim human (included in analysis), n = 10; Dalheim human (excluded from analysis), n = 5; Dalheim fauna, n = 2; negative controls, n = 4. All amplifications were performed in triplicate.

For the 18 samples that yielded consistent and high quality DNA sequences for all three genetic targets, Table 1 provides their mitochondrial HVRI sequences, and Table 2 provides the genetic sex and LP genotype results. In both tables, genetic data for the lab analysts are provided for comparison. Genetic sex was consistent with osteological sex, further authenticating the genetic results, and real-time PCR revealed asymmetrical molecular behavior consistent with authentic ancient DNA (Figure 2). We observed a 50% T-13910 allele frequency in the Dalheim samples and a total LP genotype frequency of 72%: 28% CC, 44% CT, 28% TT. These values are much higher than those previously reported in Neolithic and medieval European populations (Table 3; Figure 1). By contrast, the Dalheim results compare very closely to empirically determined T-13910 allele and LP genotype frequencies in modern-day Germany and Austria (Table 4, Figure 1). Non-European LP SNPs were not identified within our samples. The Dalheim C/T-13910 allele frequencies do not significantly differ from Hardy-Weinberg proportions (χ^2^ = 0.11, df = 2, *p* = 0.95).

**Table 1:** Consensus mitochondrial HVRI data for Dalheim human samples and laboratory analysts.

16…	051	093	163	172	182	183	184	189	192	234	311	324	342	354	356	362	368
rCRS*	A	T	A	T	A	A	C	T	C	C	T	T	T	C	T	T	T
*Dalheim samples*																	
B7			.	.	.	.	.	.	.		.	.	.	.	.	.	.
B11			.	.	.	.	.	.	.		.	.	.	.	.	.	.
B14	.	.	.	.	.	.	.	C	.	.	.	.	.	.	.	C	.
B15			.	.	.	.	.	.	.		.	.	.	.	.	.	.
B26a			.	.	.	.	.	.	.		.	.	.	.	.	.	.
B27			.	.	.	.	.	C	.		.	.	.	.	.	.	.
B30b2			.	.	.	.	.	.	.		.	.	.	.	.	.	.
B32											.	.	.	.	C	.	.
B36											.	.	.	.	.	.	C
B39	.	C	.	.	.	.	.	.	.	.	.	.	.	.	.	C	.
B40			.	.	.	.	.	C	.		.	.	.	.	.	.	.
B57			.	.	.	.	.	.	T		.	.	.	.	.	.	.
B59			.	.	.	.	.	.	.		.	.	.	.	.	.	.
B78			.	.	.	.	.	.	.		.	.	.	.	.	.	.
B82											C	.	.	.	.	.	.
B85			G	.	.	.	.	.	.		.	.	.	.	.	.	.
B85a1											.	.	.	T	.	.	.
G12											.	.	.	.	C	C	.
*Lab Analysts*																	
GA†	G	C	.	.	.	.	T	C	.	T	.	.	C	.	.	.	.
AK‡	.	.	.	.	.	.	.	.	.	.	.	.	.	.	.	C	.
AB‡	.	.	.	C	.	.	.	.	.	.	.	.	.	.	.	.	.
CW	.	.	.	.	C	C	.	C	.	T	.	C	.	.	.	.	.

*Notes:*

* Polymorphic sites are numbered according to the revised Cambridge Reference Sequence (rCRS).

Reported data consist of the consensus sequence for a minimum of two successful amplifications. A dot indicates identity to the rCRS sequence, and blank cells indicate HVRI loci for which no PCR amplification was attempted.

† Performed DNA extractions.

‡ Performed DNA amplifications.

**Table 2:** Results of genetic sex and LP allele genotyping.

	Sex^a^		C/T-13910^b^					
Individual	Osteological Sex	Genetic Sex^c^	Extraction 1		Extraction 2–3		Consensus Genotype	Inferred phenotype
			C	T	C	T		
*Dalheim samples*								
B7	I	M	18	0	4	0	CC	Non-LP
B11	M	M	5	11	1	4	CT	LP
B14	M	M	4	8	1	1	CT	LP
B15	M	M	0	8	0	13	TT	LP
B26a	*	F	18	0	4	0	CC	Non-LP
B27	I	F	10	4	2	3	CT	LP
B30b2	*	F	0	7	0	14	TT	LP
B32	I	M	14	3	23	3	CT	LP
B36	M	M	17	3	15	21	CT	LP
B39	M	M	8	7	3	22	CT	LP
B40	M	M	0	18	0	2	TT	LP
B57	F	F	16	0	3	0	CC	Non-LP
B59	M	M	18	0	4	0	CC	Non-LP
B78	F	F	9	10	16	8	CT	LP
B82	I	F	0	16	0	3	TT	LP
B85	F	F	2	12	5	7	CT	LP
B85a1	*	F	0	14	0	4	TT	LP
G12	F	F	15	0	5	0	CC	Non-LP
*Lab analysts*								
GA†							CC	Non-LP
AK‡							CC	Non-LP
AB‡							TT	LP
CW							TT	LP

* Tooth sample was collected from a mixed burial assemblage containing multiple individuals.

† Performed DNA extractions.

‡ Performed DNA amplifications.

a M, male; F, female; I, indeterminate. Osteological sex determination is from [39].

b A minimum of two independent DNA extractions were performed for each individual.

LP genotype was determined from sequence clones obtained from a minimum of two successful amplifications from Extract 1 and a minimum of one successful amplification from Extract 2 and/or Extract 3. The combined sequencing results are reported for each extract, as well as the consensus LP genotype and inferred phenotype.

c Consensus genetic sex determined from a minimum of two successful amplifications.

**Table 3:** Reported C/T-13910 genotype frequencies in ancient DNA studies.

Period	Date	Region	N	CC	CT	TT	T-13910 freq.	LP freq.	Reference
Neolithic	5500–5000 BC	Eastern Europe	8	1.00	0	0	0	0	[14]
Neolithic	5000 BC	Spain	7	1.00	0	0	0	0	[37]
Neolithic	3000 BC	France	26	1.00	0	0	0	0	[36]
Neolithic	3000–2500 BC	Basque	26	0.73	0.08	0.19	0.23	0.27	[34]
Neolithic	2800–2200 BC	Scandinavia	10	0.90	0.10	0	0.05	0.10	[35]
Medieval	AD 900–1000	Hungary	13	1.00	0	0	0	0	[38]
Medieval	AD 900–1000	Hungary	9	0.67	0.11	0.22	0.28	0.33	[38]
Medieval	AD 950–1200	Germany	18	0.28	0.44	0.28	0.50	0.72	This study

**Table 4:** Reported C/T-13910 allele frequencies in modern DNA studies of western Central Europe.

Location	N	CC	CT	TT	T-13910 freq.	LP freq.	Reference
Austria	220	0.21	0.42	0.37	0.58	0.79	[7]
Austria	490	0.29	0.51	0.20	0.45	0.71	[9]
Austria	94	0.20	0.53	0.27	0.53	0.80	[11]
Austria	258	0.24	0.49	0.28	0.52	0.76	[10]
Germany	417	0.21	0.53	0.26	0.52	0.80	[8]

## Discussion

The European T-13910 LP allele is among the few human genetic variants with evidence of strong recent selection. Nevertheless, the origin and evolutionary history of this allele remains subject to great debate. Previous studies have established that the T-13910 LP allele was largely absent from early European Neolithic farmers, including LBK populations [14], but little work has been conducted on later periods. A study of medieval Hungary found moderate levels of LP in local commoners (33%) ca. AD 900–1100, but extrapolating from these results is complicated by the region's history of conquest by lactase non-persistent Asian invaders [38]. This study of the Dalheim cemetery is the first to investigate post-Neolithic LP frequency in western Central Europe, where LP frequencies are >70% today.

Recently, it has been speculated that the major population declines associated with the AD 1346–1352 Black Death epidemics may have impacted allele frequencies in Europe either through selection or drift. Although this discussion has primarily focused on the *CCR5-Δ32* allele [40], [41], it raises the question of whether Black Death population losses, reportedly 30% of the entire European population [40], may have also played a role in altering the frequency of other alleles through drift due to population decline.

Both direct radiocarbon dating and historical records securely identify the Dalheim cemetery as a pre-Black Death cemetery, allowing pre-epidemic LP frequencies to be measured. The high frequency of C/T-13910 LP genotypes observed in the Dalheim cemetery (72%) falls within the range of LP frequencies reported for present-day Germany and Austria (71–80%) and the alleles are in Hardy-Weinberg equilibrium, suggesting allele frequency stability over the last millennium. Thus, there is no evidence of C/T-13910 LP frequency change associated with the Black Death. Additionally, our results suggest that the incomplete selective sweep posited for the T-13910 allele [24] may have reached the present allele frequency in western Central Europe by ca. AD 1200.

## Conclusion

Lactase persistence is a genetic trait enabling life-long digestion of the milk sugar lactose. The ability to rely on ruminant secondary products, such as milk, likely conveyed selective advantage during times of resource scarcity, and genetic lactase persistence has independently evolved at least five times in European, Middle Eastern, and African populations. Previous ancient DNA studies have established that genetic lactase persistence was low or absent in most European Neolithic populations. In this study, we show that the frequency of lactase persistence in medieval Germany (72%) is similar to that found today in Germany and Austria (71–80%), suggesting that the incomplete selective sweep of the lactase persistence allele may have reached the present allele frequency in western Central Europe by AD 1200. Although many aspects of the origin and early evolutionary history of the T-13910 LP allele remain uncertain, ancient DNA research has made great strides in narrowing the period of European LP selection to an approximately 4,000 year window spanning 3000 BC to AD 1200. Future ancient studies on this period are likely to reveal the specific evolutionary forces acting on the T-13910 allele and the relationship between dairying and LP genotypes.

## Materials and Methods

### Samples

The Dalheim skeletal assemblage was excavated from 1989–1990 under the direction of the Westphalian Museum of Archaeology. The assemblage consists of approximately 150 individuals recovered from a medieval cemetery attached to a parish church and convent in the village of Dalheim, located in the state of Nordrhein-Westphalen, Germany. AMS radiocarbon dating of human bone collagen indicates that this cemetery was in use for approximately 250 years, from ca. AD 950–1200 [39]. Historical records place the abandonment of the church-convent complex ca. AD 1400, following outbreaks of plague in AD 1348/1350 and AD 1383 and a late 14^th^ century battle [42]. The assemblage includes the skeletal remains of both sexes ranging in age from neonates to the elderly [39], and there is no evidence for age or sex structuring within the cemetery. In 2005, the skeletal assemblage was donated to the University of Zürich's Institute of Anatomy for scientific investigation [39]. The handing history of the assemblage prior to its arrival at the University of Zürich is not well documented, but appears to have been minimal. Dentine samples from 36 randomly selected adults were collected for genetic analysis. No permits were required for the described study, which complied with all relevant regulations.

### Contamination prevention

All DNA extractions were performed in a dedicated ancient DNA laboratory facility at the University of Zurich's Centre for Evolutionary Medicine. The Ancient DNA Laboratory, composed of four self-contained rooms with an independent HEPA air filtration system, is dedicated solely to ancient DNA research and follows established contamination control workflows, including physical separation from all laboratories in which PCR is performed, unidirectional work flows to avoid cross-contamination, regular sterilization of all work surfaces with 1–2% sodium hypochlorite (NaOCl) solution, overhead UV lights for daily air and surface decontamination, and the use of full body Tyvek suits, masks, and gloves by all researchers. Reagents used for amplification were additionally decontaminated using a combination of UV irradiation and reagent pretreatment with heat labile double-stranded DNAse (ArcticZymes, Norway) to purify dNTPs, primers, BSA, and enzymes [43]. Within the Ancient DNA Laboratory, sample collection was performed in a dedicated sample preparation area containing an additional HEPA-filtered vacuum system, and all sensitive aspects of DNA extraction and PCR set-up were performed within HEPA-filtered laminar flow hoods located in separate rooms within the laboratory dedicated to extraction and PCR set-up. Negative controls (non-template extraction controls and reagent blanks) were processed in parallel with all samples and were continuously monitored for contamination. All PCR amplification and post-PCR analyses were performed in physically separated laboratory facilities. Although there are no perfect criteria for contamination exclusion and ancient DNA authentication [44], we have attempted to use prudence in designing the following experiments, in self-critically evaluating contamination sources and risks, and in developing clear and objective criteria for sample inclusion and exclusion.

### DNA Extraction

The tooth material was cleaned with sterile single use towels soaked with 2% NaOCl to remove any surface contamination and then soaked in a small volume of DNA-free water (molecular biology grade, DNAse-free, and UV-irradiated) to remove remaining NaOCl. Dentine was pulverized using a SPEX freezer mill 6770, and mill cartridges were cleaned with a 2% NaOCl solution between samples. Approximately 100 mg of dentine powder was digested overnight in 1 ml of a 0.45 M EDTA and 10% Proteinase K (Qiagen) buffer at 55°C, and then for an additional 24 hours on a rotating nutator at room temperature. After centrifugation, the supernatant was extracted twice with a phenol, chloroform and isoamyl alcohol mixture (25∶24∶1), followed by a final chloroform step. QiaQuick PCR purification columns were used to isolate the DNA, which was eluted into 30 µl of EB buffer twice following manufacturer's instructions. At least two independent DNA extractions were performed for each individual, and two non-template control extractions were performed in parallel for every twelve samples. The DNA yield of 1 µl of extract was measured with a Qubit fluorometer high sensitivity assay. The non-template extraction controls contained no detectable amounts of DNA.

### mtDNA analysis

To exclude laboratory analysts as a potential source of human DNA contamination, we PCR-amplified one or more overlapping fragments of the mitochondrial hypervariable region I (HVRI) of the Dalheim samples in duplicate and compared the resulting sequences to analyst mtDNA HVRI sequences. The primer positions and sequences were as follows: Primer Set 1, 16288–16423 (136 bp; 98 bp internal), 1F 5′-TACCCACCCTTAACAGTACA-3′, 1R 5′-TATTGATTTCACGGAGGA-3′; Primer Set 2, 16206–16346 (141 bp, 99 bp internal), 2F 5′-AAGTACAGCAATCAACCCTC-3′, 2R 5′-CTGTAATGTGCTATGTACGGTA-3′; Primer Set 3, 16112–16247 (136 bp; 96 bp internal), 3F 5′-CACCATGAATATTGTACGGT-3′, 3R 5′-TTGCAGTTGATGTGTGATAG-3′; Primer Set 4, 16017–16179 (163 bp; 125 bp internal), 4F 5′-TTCTCTGTTCTTTCATGGG -3′, 4R 5′-GATGTGGATTGGGTTTTTA- 3′. The PCR reactions were set up as follows: 0.2 µl Phusion Hot Start II polymerase, 4 µl 5X Phusion HF buffer, 2 µl of 2 mM dNTPs, 1 µl of 2.5 mg/mL BSA, 1 µl of 10 µM forward primer, 1 µl of 10 µM reverse primer, and 9.8 µl H_2_O, plus 1 µl of sample extract. Cycling conditions were performed as follows: enzyme activation at 98°C for 30 s, followed by 46 cycles of denaturation at 98°C for 10 s, annealing at 54°C for 20 s, and elongation at 72°C for 15 s, and ending with 72°C for 10 min. Successful amplicons were analyzed by Sanger sequencing on an ABI 3730xl instrument (GATC Biotech) and aligned to analyst HVRI sequences and the revised Cambridge Reference Sequence (rCRS [45]; NC_012920.1) using CLC Main Workbench 5 (v.5.7.1) software. The HVRI sequence for each Dalheim sample was distinct from analyst sequences. Because mtDNA occurs at greater copy number than nuclear DNA and is known to have greater preservation in ancient samples, any extract that did not amplify the mtDNA target in at least two independent reactions and yield consistent results was excluded from further analysis.

### Genetic sex typing

As an additional test of DNA authenticity we performed genetic sex typing on the Dalheim samples and compared the results to osteological sex determinations for each individual. Genetic sex was assessed using a quantitative PCR (qPCR) TaqMan duplex assay (Applied Biosystems) to detect X (112 bp) and Y (106 bp) chromosome sequences of the amelogenin gene [46]. In brief, the primer sequences were: forward, 5′- CCCTGGGCTCTGTAAAGAATAGTG-3′, reverse, 5′-ATCAGAGCTTAAACTGGGAAGCTG -3′. The MGB probe sequences were FAM-TATCCCAGATGTTTCTC and VIC-CATCCCAAATAAAGTG. Real-time PCR set up was as follows: 7.3 µl LightCycler 480 Probes Master Mix, 0.4 µl 40× Custom Genotyping Assay (each primer 36 µM, each probe 8 µM, Applied Biosystems), 6.3 µl H_2_O, and 1 µl sample extract. Real-time PCR was performed on a Roche LightCycler 480 with the following cycling conditions: enzyme activation at 95°C for 10 min, followed by 60 cycles of denaturation at 95°C for 15 s and annealing and extension at 58°C. Each extract was analyzed a minimum of six times, and three non-template extraction controls and three reagent blanks were processed in parallel with each qPCR run. No amplification was observed in any non-template extraction control or reagent blank. Any extract that failed to amplify the amelogenin locus at least three times was excluded from C/T-13910 genotype analysis.

### LP genotyping

LP genotypes were assessed by PCR-amplifying a 111 bp target [14] containing four LP SNPs (C/G-13907, C/T-13910, T/C-13913, T/G-13915), followed by cloning and sequencing (Table 2). Because cytosine deamination damage at the site of the primary SNP of interest (C/T-13910) could result in base misincorporation during PCR using a conventional Taq-based enzyme and therefore falsely increase our estimate of the derived allele, we chose to use Phusion, a high fidelity DNA polymerase that fails to amplify damaged DNA [47], resulting in more accurate SNP typing. The PCR reaction was set up as follows: 0.2 µl Phusion Hot Start II polymerase, 4 µl 5X Phusion HF buffer, 2 µl of 2 mM dNTPs, 1 µl of 2.5 mg/mL BSA, 1 µl of 10 µM forward primer 5′-GCGCTGGCAATACAGATAAGATA-3′, 1 µl of reverse primer 5′-AATGCAGGGCTCAAAGAACAA-3′, and 9.8 µl H_2_O, plus 1 µl of sample extract. Cycling conditions were performed as follows: enzyme activation at 98°C for 30 s, followed by 48 cycles of denaturation at 98°C for 10 s, annealing at 58°C for 20 s, and elongation at 72°C for 15 s, and ending with 72°C for 5 min. Samples that failed to yield successful amplicons from at least two extractions were excluded from analysis. Successful amplicons were cloned into pBluescript KS vectors (Stratagene) following manufacturer instructions and then transformed into competent *Escherichia coli* cells using standard protocols. For each sample, at least ten colonies from a minimum of two amplifications from the first extraction and at least five colonies from one or more amplifications of a subsequent extraction were randomly picked and PCR amplified. The PCR reaction was set up as follows: 0.2 µl GoTaq Polymerase (Promega), 5 µl 5X Green GoTaq Flexi buffer, 0.5 µl of 10 mM dNTPs (Thermo Scientific), 2.5 µl of 25 mM MgCl_2_, 1 µl of 10 mM T3 primer, 1 µl of 10 mM T7 primer, and 14.8 µl of PCR grade H_2_O. Cycling conditions were performed as follows: enzyme activation at 95°C for 10 min, followed by 20 cycles of denaturation at 95°C for 30 s, annealing at 50°C for 30 s, and elongation at 72°C for 30 s, and ending with 72°C for 10 min. Successful amplicons were analyzed by Sanger sequencing ABI 3730xl instrument (GATC Biotech). The resulting sequences were aligned to the human reference genome (GRCh37.p5) using CLC Main Workbench 5 (v.5.7.1) software and genotyped for each LP allele. LP genotypes of the lab analysts are also provided in Table 2.

### Ancient DNA authentication

In order to further authenticate our results, we tested the Dalheim samples for appropriate asymmetrical molecular behavior consistent with ancient DNA. Ancient DNA is known to be highly fragmented to sizes less than 500 bp [48], and in many cases is less than 250 bp in length. Additionally, shorter fragments are generally found in excess compared to longer fragments, resulting in asymmetrical amplification efficiency based on target length [35], [49].

To test for asymmetrical molecular behavior supporting ancient DNA authenticity, we used real-time PCR to amplify the human LP allele region using four primer pairs targeting increasing template lengths from a subset of Dalheim samples and control samples. The forward primer (same as above) was held constant, while the reverse primers were designed to generate small (62 bp, 5′-AGGAGGAGAGTTCCTTTGAGG-3′), medium (111 bp, same as above), long (170 bp, 5′-ATGCCCTTTCGTACTACTCCC-3′), and very long (441 bp, 5′-ACTTCAGGGGAAGAGGGCTA-3′) amplicons. If the DNA present in the samples is authentically ancient, the short primer pair is expected to amplify more efficiently than the medium and long primer pairs, and no amplification is expected for the very long primer pair. We tested these primer pairs on a subset of the Dalheim human samples analyzed in this study (n = 10; B7, B11, B14, B26a, B27, B30b2, B36, B40, B57, and B59), faunal controls recovered from the same site (sheep dentine, n = 2), and negative controls (non-template reagent blanks, n = 4). Additionally, we also analyzed a subset of Dalheim human samples (n = 5; B17, B34, B43, B50, and B52) that were excluded from this study for failing to meet minimal DNA preservation thresholds.

All samples and controls were analyzed in triplicate by SYBR Green assay using a LightCycler 480 real-time PCR instrument (Roche). The 20 µl real-time PCR reaction was set up as follows: 10 µl LightCycler 480 SYBR Green I Master Mix, 1 µl of 10 µM forward primer, 1 µl of reverse primer, and 7 µl H_2_O, plus 1 µl of sample extract. Cycling conditions were performed as follows: pre-incubation at 95°C for 5 minutes, followed by 55 cycles of denaturation at 95°C for 10 s, annealing at 60°C for 15 s, and elongation/SYBR Green acquisition at 72°C for 20 s. After amplification, the melting curve was determined according to manufacturer instructions. Successful amplifications and amplification artifacts (e.g., primer dimers) were also visualized and evaluated using gel electrophoresis. Comparisons between samples were made on the basis of amplification success and relative template abundance inferred by Cp value.

The results of the real-time PCR analysis conform to expectations of asymmetrical molecular behavior for ancient DNA and support the authenticity of the Dalheim human ancient DNA results (Figure 2). For all four primer pairs, no product was observed in the negative control amplifications (0/48), despite the fact that the real-time PCR was allowed to continue to 55 cycles. This confirms that our decontamination precautions have resulted in a highly clean laboratory environment. Real-time PCR of the Dalheim faunal controls resulted in one positive amplification (1/24) of the shortest human primer pair at a Cp value of 40.7. Subsequent visualization by gel electrophoresis confirmed the presence of a faint band, but there was insufficient PCR product for sequencing. This result indicates that prior handing of the Dalheim skeletal collection has resulted in trace human contamination; however, the fact that this contamination amplified at a late cycle, yielded insufficient product for sequencing, and could not be replicated indicates that this contamination poses minimal risk to the results of this study.

In contrast to the controls, the Dalheim human samples analyzed in this study showed good DNA preservation with high amplification replicability and asymmetrical molecular behavior consistent with authentic ancient DNA. All amplifications were successful for the short 62 bp DNA target (30/30), and nearly all amplifications were successful for the medium length 111 bp DNA target (28/30) used for allele genotyping in this study. However, amplification was much less successful for the long 170 bp DNA target (18/30), and no amplification was observed for the very long 441 bp DNA target (0/30). This inverse relationship between successful DNA amplification and target length is consistent with known patterns of ancient DNA fragmentation. Additionally, the mean Cp value for successful amplifications was 35.6±1.4, indicating a relatively high number of starting template molecules per PCR reaction, and the highest Cp value was 39.7.

For comparison, we also analyzed a subset of Dalheim human samples that had been excluded from the study because they were found to have a high amplification failure rate during mtDNA analysis, sex typing, and LP genotyping. Real-time PCR analysis revealed that these samples also exhibited asymmetrical molecular behavior consistent with ancient DNA, but their amplification success rates were much lower, a result consistent with our earlier observations. Thus, while these samples likely also contain authentic ancient DNA, their DNA quantity and/or quality is lower, making them less reliable sources of genotyping data and more susceptible to amplification bias and allelic dropout. By excluding these samples and instead relying on only well-preserved ancient human samples, we reduce the risk of potential bias, artifacts and contamination in our data.

### Statistical analysis

A statistical evaluation of Hardy Weinberg equilibrium was assessed by a Pearson's Chi-squared test of observed and expected genotype data in 3×2 contingency table using R statistical software (v. 3.0.1) [50].
